# Feedforward object-vision models only tolerate small image variations compared to human

**DOI:** 10.3389/fncom.2014.00074

**Published:** 2014-07-18

**Authors:** Masoud Ghodrati, Amirhossein Farzmahdi, Karim Rajaei, Reza Ebrahimpour, Seyed-Mahdi Khaligh-Razavi

**Affiliations:** ^1^Brain and Intelligent Systems Research Laboratory, Department of Electrical and Computer Engineering, Shahid Rajaee Teacher Training UniversityTehran, Iran; ^2^School of Cognitive Sciences, Institute for Research in Fundamental Sciences (IPM)Tehran, Iran; ^3^Department of Physiology, Monash UniversityMelbourne, VIC, Australia; ^4^Department of Electrical Engineering, Amirkabir University of TechnologyTehran, Iran; ^5^MRC Cognition and Brain Sciences Unit, University of CambridgeCambridge, UK

**Keywords:** computational model, invariant object recognition, reaction time, object variation, visual system, feedforward models

## Abstract

Invariant object recognition is a remarkable ability of primates' visual system that its underlying mechanism has constantly been under intense investigations. Computational modeling is a valuable tool toward understanding the processes involved in invariant object recognition. Although recent computational models have shown outstanding performances on challenging image databases, they fail to perform well in image categorization under more complex image variations. Studies have shown that making sparse representation of objects by extracting more informative visual features through a feedforward sweep can lead to higher recognition performances. Here, however, we show that when the complexity of image variations is high, even this approach results in poor performance compared to humans. To assess the performance of models and humans in invariant object recognition tasks, we built a parametrically controlled image database consisting of several object categories varied in different dimensions and levels, rendered from 3D planes. Comparing the performance of several object recognition models with human observers shows that only in low-level image variations the models perform similar to humans in categorization tasks. Furthermore, the results of our behavioral experiments demonstrate that, even under difficult experimental conditions (i.e., briefly presented masked stimuli with complex image variations), human observers performed outstandingly well, suggesting that the models are still far from resembling humans in invariant object recognition. Taken together, we suggest that learning sparse informative visual features, although desirable, is not a complete solution for future progresses in object-vision modeling. We show that this approach is not of significant help in solving the computational crux of object recognition (i.e., invariant object recognition) when the identity-preserving image variations become more complex.

## Introduction

The beams of light reflecting from visual objects in the three-dimensional natural environment provide two-dimensional images onto the retinal photoreceptors. While the object is the same, an infinite number of light patterns can be mirrored in the retinal photoreceptors depending on object's distance (size), position, lightening condition, viewing angle (in-depth or in plane), and background. Therefore, the probability of having the same image on retina generated by an identical object in two different times, even in successive frames that are temporally close, is quite close to zero (DiCarlo and Cox, 2007; Cox, 2014). However, the visual system outstandingly performs object recognition, accurately and swiftly, despite substantial transformations.

The human brain can recognize the identity and category membership of objects within a fraction of a second (~100 ms) after stimulus onset (Thorpe et al., 1996; Carlson et al., 2011; Baldassi et al., 2013; Isik et al., 2013; Cichy et al., 2014). The mechanism of this remarkable performance in the unremitting changes of visual conditions in the natural world has constantly been under intense investigations, both experimentally and computationally (reviewed in Peissig and Tarr, 2007; DiCarlo et al., 2012; Cox, 2014). Our visual system can discriminate two highly similar objects within the same category (e.g., face identification) in various viewing conditions (e.g., changes in size, pose, clutter, etc.—invariance). However, this task is a very complex computational problem (Poggio and Ullman, 2013).

It is thought that the trade-off between selectivity and invariance is evolved through hierarchical ventral visual stages starting from the retinal to the lateral geniculate nucleus (LGN), then through V1, V2, V4, and finally IT cortex (Kreiman et al., 2006; Zoccolan et al., 2007; Rust and DiCarlo, 2010, 2012; Sharpee et al., 2013). Decades of investigations on the visual hierarchy have shed light on several fundamental properties of neurons in the ventral visual stream (Felleman and Van Essen, 1991; Logothetis and Sheinberg, 1996; Tanaka, 1996; Cox, 2014; Markov et al., 2014). We now know that neurons in the higher level visual areas, such as IT, have larger receptive fields (RFs) compared to the lower levels in the hierarchy (e.g., V1). Each higher level neuron receives inputs from several neurons in the lower layer. Therefore, up-stream neurons in the hierarchy are expected to respond to more complex patterns such as curvature for V4 neurons (reviewed in Roe et al., 2012) and objects for IT neurons compared to the early visual areas, which are responsive to bars and edges (Carandini et al., 2005; Freeman et al., 2013).

Using a linear read-out method, Hung et al. (2005) were able to decode the identity of objects from neural activities in primate IT cortex while the size and position of objects varied. This shows that representations of objects in IT are invariant to changes in size and position. Moreover, recent studies have reported intriguing results about object recognition in various stages and times in the ventral visual stream using different recording modalities in different species (e.g., Haxby et al., 2001; Hung et al., 2005; Kiani et al., 2007; Kriegeskorte et al., 2008b; Freiwald and Tsao, 2010; Cichy et al., 2014). Nevertheless, the mechanism of invariant object recognition has remained unknown to a certain extent. Most studies that have attempted to address invariant object recognition have used objects with gray backgrounds while either frontal views of objects were presented or only simple objects with limited variations were used (e.g., Alemi-Neissi et al., 2013; Isik et al., 2013; Wood, 2013). Studying the underlying computational principles of invariant object recognition is a very complicated problem with many confounding factors such as complex variations in real-world objects that makes it even more abstruse. This may explain why in most studies more attention is paid to understanding object recognition under restricted conditions by disregarding these complex variations from the stimulus set.

Recent recording studies have evidenced that representations of objects in IT are more invariant to changes in object appearance than intermediate levels of the visual ventral stream, such as V4 (Yamins et al., 2014). This shows that invariant representations are evolving across the visual hierarchy. Modeling results, inspired by biology, have also demonstrated that a great level of invariance is achievable using several processing modules built upon one another in a hierarchy from simple to complex units (e.g., Wallis and Rolls, 1997; Riesenhuber and Poggio, 1999; Rolls, 2012; Anselmi et al., 2013; Liao et al., 2013).

Computational modeling is a valuable tool for understanding the processes involved in biological object vision. Although recent computational models have shown outstanding performances on challenging natural image databases (e.g., Mutch and Lowe, 2006; Serre et al., 2007b; Ghodrati et al., 2012; Rajaei et al., 2012) and compared to human (Serre et al., 2007a), they fail to perform well when they are presented with object images under more complex variations (Pinto et al., 2008). It has also been shown that the representations of object categories in object-vision models are weakly correlated with human and monkey IT cortex (Kriegeskorte, 2009; Kriegeskorte and Mur, 2012; Khaligh-Razavi and Kriegeskorte, 2013). This may explain why models do not yet achieve human level of categorization performance. Some studies have suggested that instead of a random sampling of visual features (Serre et al., 2007a), extracting a handful of informative features can lead to higher recognition performances (Ullman et al., 2002; Ghodrati et al., 2012; Rajaei et al., 2012). Having said that, we show in this study that when image variations are high, yet this approach results in poor performances compared to humans. Furthermore, we also show that the models do not form a strong categorical representation when the image variation exceeds a threshold (i.e., objects in the same category do not form a cluster in higher levels of variations).

Here we compare the performance of several object recognition models (Mutch and Lowe, 2006; Serre et al., 2007a; Pinto et al., 2008; Ghodrati et al., 2012; Rajaei et al., 2012) in invariant object recognition. Using psychophysical experiments, we also compare the performance of the models to human observers. All models are based on the theory of feedforward hierarchical processing in the visual system. Therefore, to account for the feedforward visual processing, images in our psychophysical experiments were rapidly presented to human observers (25 ms) followed by a mask image. As a benchmark test we also evaluated the performance of one of the best known feedforward object recognition models (Krizhevsky et al., 2012) against humans to see how far the best performing object-vision models go in explaining profiles of human categorization performance.

We employed representational similarity analysis (RSA), which provides a useful framework for measuring the dissimilarity distance between two representational spaces independent of their modalities (e.g., human fMRI activities and models' internal representations—see Kriegeskorte et al., 2008a; Kriegeskorte, 2009). In this study we used RSA to compare the representational geometry of the models with that of the human observers in invariant object recognition tasks.

To evaluate the categorization performance of the models and humans we built a parametrically controlled image database consisting of different object categories, considering various object variations, rendered from 3D planes (O'Reilly et al., 2013). Generating such controlled variations in object images helps us to gain better insights about the ability of models and humans in invariant object recognition. It also helps experimentalists to study invariant object recognition in human and monkey by taking advantage of having controlled variations over several identity-preserving changes of an object.

Our results show that human observers have remarkable performances over different levels of image variations while the performances of the models were only comparable to humans in the very first levels of image variations. We further show that although learning informative visual features improves categorization performance in less complex images (i.e., images with fewer confounding variations), it does not help when the level of confounding variations (e.g., variations in size, position, and view) increases. The results of our behavioral experiments also demonstrate that models are still far from resembling humans in invariant object recognition. Moreover, as the complexity level of object variations increases (from low to intermediate and high levels of variations), models' internal representation become worse in disentangling the representation of objects that fall in different categories.

## Materials and methods

### Image generation process

One of the foremost aspects of the evaluation procedure described in this study is the utilization of controlled variations applied to naturalistic objects. To construct various two-dimensional object images with controlled variations, we used three-dimensional meshes (O'Reilly et al., 2013). It allowed us to parametrically control different variations, background, number of objects in each class, etc. Therefore, we were able to parametrically introduce real-world variations in objects.

For each object category (car, motorcycle, animal, ship, airplane), we had on average sixteen 3D meshes (showing different exemplars for each category) in which 2D object images were rendered using rendering software with a uniform gray background for all images. Throughout the paper we call them objects on plain backgrounds. These images were superimposed on randomly selected backgrounds from a set of more than 4000 images (see Figure S1 for image samples with natural backgrounds). The set included images from natural environments (e.g., forest, mountain, desert, etc.) as well as man-made environments (e.g., urban areas, streets, buildings, etc.). To preserve a high variability in our background images, we obtained all background images using the internet.

**Figure 1:**
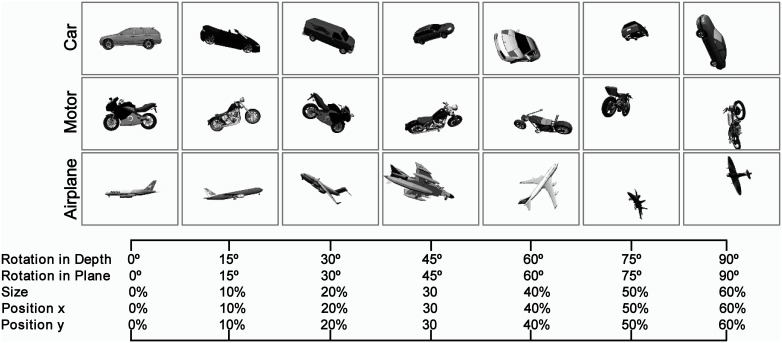
**Sample images in different levels of variation with Plain Background**. The Object images, rendered from 3D planes, vary in four dimensions: size, position (x, y), rotation in-depth, and rotation in plane. To alter the complexity of the images, we constructed images in seven levels of variations starting from zero level variation, which no variation is applied to 3D object planes (first column at left), to seventh level of variation, which substantial variations are applied to images (last column at right). In each level of variation, we randomly sample different values for each dimension (e.g., size, rotation, and position) from a uniform distribution and finally selected values are applied to a 3D plane. As the level of variation increases, the range of values increases. There are several sample object images with natural background in the supplementary materials (Figure S1).

Naturalistic object images were varied in four different dimensions: position (across x and y axes), scale, in-depth rotation, and in-plane rotation (Figure 1). To alter the difficulty of the images and tasks, we used seven levels of variation to span a broad range of diversity in the image dataset (starting from no particular variations, Figure 1-left, to the intermediate and complex image variations, Figure 1-right). The amount of object transformations in each level and dimension was selected by random sampling from a uniform distribution. For example, to generate images with second level of variation (i.e., Level 1), we randomly sampled different degrees for in-depth rotation (or in-plane rotation) from a range of 0–15° using a uniform random distribution. The same sampling procedure was applied to other dimensions (e.g., size and position). Then, these values were applied to a 3D mesh and a 2D image was subsequently generated from the 3D mesh. A similar approach was taken for generating images in other levels of variation.

### Psychophysical experiment

Two experiments were designed to investigate the performance of human subjects in invariant object recognition: tow- and multiclass invariant object categorization task.

#### Two-class invariant object categorization

In total, 41 subjects (24 male, age between 21–32, mean age 26) participated in the first experiment. We used 560 object images (300 × 400 pixels, grayscale images) selected from seven levels of variation and two different object categories (80 images for each level with 40 images from each category) for each session. Images were presented on a 21″ CRT monitor with a resolution of 1024 × 724 pixels and a frame rate of 80 Hz. We used Matlab with the Psychophysics Toolbox to present the images (Brainard, 1997; Pelli, 1997). The viewing distance was 60 cm.

**Figure 2:**
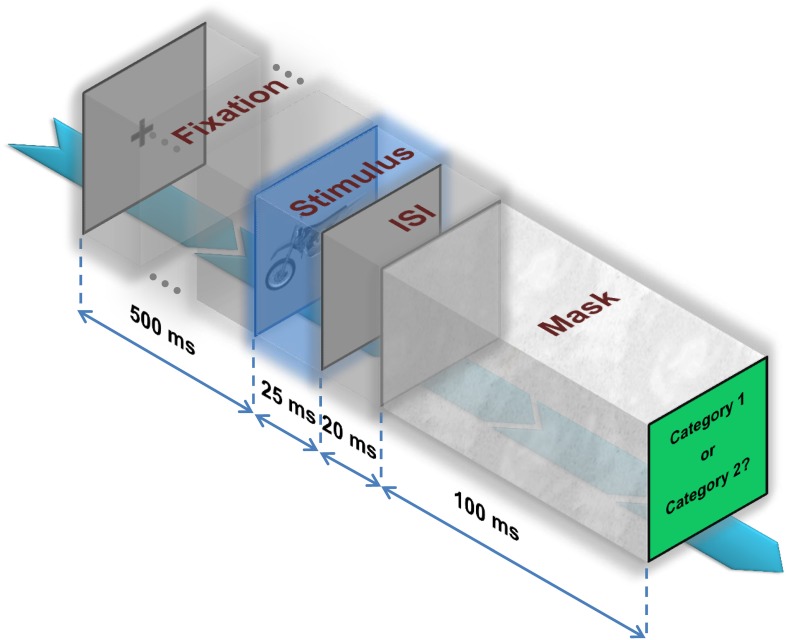
**Psychophysical experiment**. A fixation cross is presented for 500 ms. Then, an image is randomly selected from the dataset and presented at the center of the screen for the duration of 25ms. Subsequently, a blank screen is presented on the screen for the duration of 20 ± 2 ms (interstimulus interval—ISI) followed by a mask image that stays on the screen for 100ms. Finally, subjects have to decide whether the image belongs to category 1 or 2.

Following a fixation cross, which was presented for 500 ms, an image was randomly selected from the dataset (considering levels and categories) and presented at the center of the screen for the duration of 25 ms. Subsequently, a blank screen was presented for the duration of 20 ± 2 ms (interstimulus interval-ISI) and a mask image was presented after the blank screen and stayed on for 100 ms (Figure 2). The mask image was a (1/f) random noise.

Subjects were instructed to complete four sessions (cars vs. animals, cars vs. motors, with plain and natural background). Some subjects completed all four sessions and some only finished some sessions. In each session, 560 images (e.g., 280 cars and 280 motors) were presented in a random order and were divided into 4 blocks of 140 images each. There was a time interval of 5 min between blocks for each subject to take a rest. The reaction times (RTs) of participants were recorded to investigate whether there is any time difference in categorization between levels and categories.

The subjects' task was to determine whether the presented image was a car or a/an motor/animal by pressing “C” or “M” on a computer keyboard, respectively. Keys were labeled on the keyboard with the name of corresponding categories. Subjects performed several training trials, with different images, to become familiar with the task prior to the actual experiment. In training trials (30 images), a sentence was presented on the monitor showing whether the answers were correct or not. During the main procedure, the participants had to declare their decision by pressing the keys; but no feedback was given to them regarding the correctness or incorrectness of the choices. The next trial was instantly started after getting subject's response. Subjects were instructed to respond as fast and accurate as possible to the presented image. All subjects voluntarily accepted to participate in the task and gave their written consent.

#### Multiclass invariant object categorization

In total, 26 subjects participated in the second behavioral experiment (17 male, age between 21–32, mean age of 26 years). Object images were selected from five categories (i.e., car, animal, motorcycle, ship, and airplane) in seven levels of variation. The procedure was the same as the first experiment: an image was randomly selected and presented on the center of the screen for 25 ms after a fixation cross (500 ms). Subsequently, a blank screen (ISI) of 20 ± 2 ms was presented followed by a mask image, which stayed on for 100 ms (Figure 2). Subjects were instructed to indicate the image category by pressing one of the five keys on the computer keyboard, each labeled with a name representing a specific category (“C,” “Z,” “M,” “N,” and “/” for car, animal, motorcycle, ship, and airplane, respectively). The next trial was started by pressing the space-bar. The RTs of subjects were not evaluated in this task, so subjects had time to state their decisions. However, subjects were instructed to respond as fast and accurately as possible.

This task was designed to have two sessions (images with plain and natural background). In each session, 700 images (100 images per level, 20 images from each object class in each level) were presented in a random order, divided into 4 blocks of 175 images each. There was a gap of 5 min between blocks for subjects to take a rest. Some subjects completed all sessions and some only finished some of them. Subjects performed a few example trials before starting the actual experiment (none of the images in these trials were presented in the main experiment). In training trials (30 images), a sentence was presented on the monitor as a feedback showing the correctness/incorrectness of the answers. In the main procedure, participants had to declare their decision by pressing one of the keys; but no feedback was given to them regarding the correctness of choices. All subjects voluntarily accepted to participate in the task and gave their written consent.

### Human representational dissimilarity matrix (RDM)

In the multiclass psychophysical experiment, subjects' responses to the presented stimuli were recorded. Subjects had five choices for each presented stimulus: 1–5 for five categories. We constructed a matrix, R, based on the subjects' responses. The rows of R were labels assigned to an image by different subjects (each row corresponds to one image) and each column contained responses of one subject to all images in the task. Therefore, the size of this matrix was: images × subjects (e.g., for the multiclass experiment the size was 700 × 17 for each task, plain and natural background). Afterwards, we calculated the categorization score for each row of the matrix. To do this, for example, out of 17 participants (e.g., responses in row one), 11 selected category one for the presented image, five responses showed category two, and one classified the image as category three, and no subject classified the image as category four and five. This gives us a response pattern (R_*1,1:5*_) for the first image (e.g., the image in the first row):

$${\text{R}}_{1,1:5}=\left[11\;5\;1\;0\;0\right]$$

Finally, we normalized each row by dividing it to the number of responses:

$${\text{R}}_{1,1:\;5}=\frac{\left[11\;5\;1\;0\;0\right]}{17}=\left[0.6471\;0.2941\;0.0588\;0\;0\right]$$

To calculate the RDMs, we used the RSA toolbox developed by Nili et al. (2014). Each element in a given RDM shows the pairwise dissimilarity between the response patterns elicited by two images. RDM is a useful tool to visualize patterns of dissimilarities between all images in a representational space (e.g., brain or model). The dissimilarity between two response patterns is measured by correlation distance (i.e., 1-correlation—here we used Spearman's rank correlation). RDMs are directly comparable to each other and they provide a useful framework for comparing the representational geometry of the models with that of the human independent of the type of modalities and represented features (e.g., human behavioral scores and models' internal representations).

### Computational models

#### V1-like

This model is a population of simple and complex cells fed by luminance images as input. We used Gabor filters at four different orientations (0, 45, 90, and −45°) and 12 sizes (7–29 pixels with steps of two pixels) to model simple cell RFs. Complex cells were made by performing the MAX operation on the neighboring simple cells with similar orientations. The outputs of all simple and complex cells were concatenated in a vector as the V1 representational pattern of each image.

#### HMAX

The HMAX model, developed by Serre et al. (2007a), has a hierarchical architecture inspired by the well-known simple to complex cells model of Hubel and Wiesel (1962, 1968). The HMAX model that is used here adds two more layers (S2, C2) on the top of the complex cell outputs of the V1 model described above. The model has alternating S and C layers. S layers perform a Gaussian-like operation on their inputs, and C layers perform a max-like operation, which makes the output invariant to small shifts in scale and position. We used the freely available version of the HMAX model (http://cbcl.mit.edu/software-datasets/pnas07/index.html). The HMAX C2 features were used as the HMAX representation.

#### GMAX

GMAX is an extension of the HMAX model for which in the training phase, instead of selecting a pool of random patches, patches that are more informative for the classification task are selected. The model uses an optimization algorithm (i.e., genetic algorithm) to select informative patches from a very large pool of random patches (Ghodrati et al., 2012). In the training phase of the GMAX model the classification performance is used as the fitness function for the genetic algorithm. A linear SVM classifier was used to measure the classification performance. To run this model we used the same set of model parameters suggested in Ghodrati et al. (2012).

#### Stable

Stable model is a bio-inspired model with a hierarchy of simple to complex cells. The model uses the adaptive resonance theory (ART-Grossberg, 1976) for extracting informative intermediate level visual features. This has made the model stable against forgetting previously learned patterns (Rajaei et al., 2012). Similar to the HMAX model it extracts C2-like features, except that in the training phase it only selects the highest active C2 units as prototypes that represent the input image. This is done using top-down connections from C2 layer to C1 layer. The connections match the C1-like features of the input image to the prototypes of the C2 layer. The matching degree is controlled by a vigilance parameter that is fixed separately on a validation set. We set the model parameters the same as were suggested in Rajaei et al. (2012).

#### SLF

This is a bio-inspired model based on the HMAX C2-features. The model introduces sparsified and localized intermediate-level visual features (Mutch and Lowe, 2008). We used the Matlab code freely available for these feature (http://www.mit.edu/~jmutch/fhlib); and the default model parameters were used.

#### Pixel

The pixel representation is simply a feature vector containing all pixels of an input image. Each image was converted to grayscale and then unrolled as a feature vector. We used pixel representation as our baseline model.

#### Convolutional neural networks

Convolutional neural networks (CNNs) are bio-inspired hierarchical models of object-vision that are made of several convolutional layers (Jarrett et al., 2009). Convolutional layers scan the input image inside their RFs. RFs of convolutional layers get their input from various places in the input image, and RFs with identical weights make a unit. The outputs of each unit make a feature map. Convolutional layers are usually followed by subsampling layers that perform a local averaging and subsampling, which make the feature maps invariant to small shifts (LeCun and Bengio, 1998). In this study we used the deep supervised convolutional network by Krizhevsky et al. (2012; Donahue et al., 2013). The network is trained with 1.2 million labeled images from ImageNet (1000 category labels), and has eight layers: five convolutional layers, followed by three fully connected layers. The output of the last layer is a distribution over the 1000 class labels. This is the result of applying a 1000-way softmax on the output of the last fully connected layer. The model has 60 million parameters and 650,000 neurons. The parameters are learnt with stochastic gradient descent. The results for the deep ConvNet are discussed in Supplementary Material.

### Model evaluation

**Figure 3:**
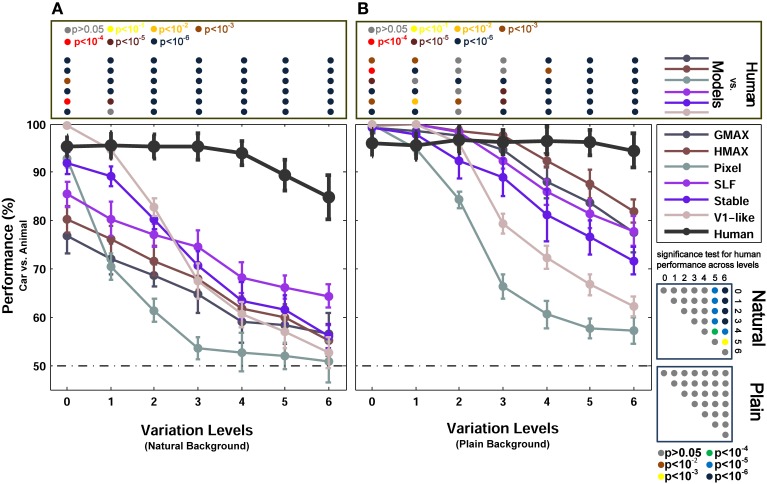
**Performances of models and human in two-class (car/animal) invariant object categorization task. (A)** Performance comparison when objects were presented on natural backgrounds (performances are the ratio of correct responses). The dashed, horizontal line shows the chance level and each curve represents the performance of a model in different levels of variation, specified with different colors at the right inset. The bold, black curve illustrates human performance. The color-coded circle points at the top of each plot, inside the rectangular box, exhibits the *p*-values for comparisons between human and each model obtained by Wilcoxon signed-rank test (for example the performance of the HMAX model was compared to the human in each level of variation separately. The result of comparison for each model in each level provides us with a *p*-value. *P*-values are reported with different colors). The color-coded circle points at the right insets, inside the square boxes, show the *p*-values for all possible comparisons between human responses in different levels of variation (with plain and natural background). Here, the *p*-values show whether human categorization performances are significantly different at different levels of variation. For example, we compared the performance of human in Level 0, with Level 1, Level 2, and so on and reported a *p*-value for each comparison. These comparisons resulted in a symmetric *p*-value matrix with the size of 7^*^7 (i.e., 7 levels of variations). **(B)** Performance comparison when objects were presented on plain backgrounds. In both panels **(A,B)**, the results are the average of 15 independent random runs and the error bars show the standard deviation of the mean.

**Figure 4:**
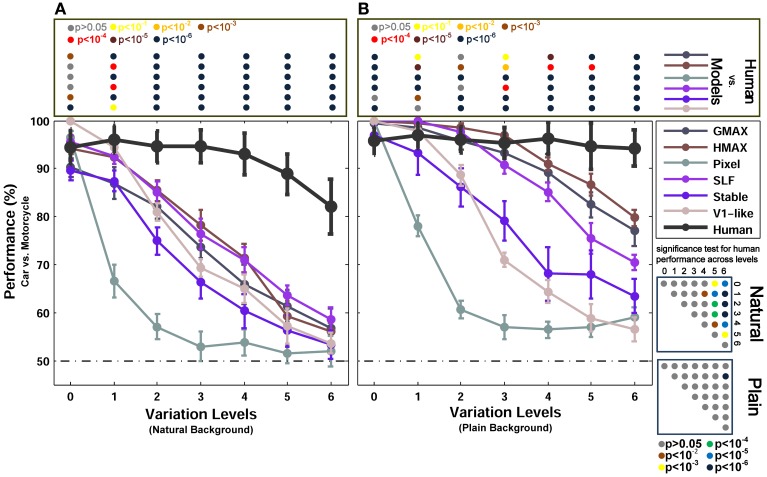
**Performances of models and human in two-class (motorcycle/car) invariant object categorization task. (A)** Performance comparison when objects were presented on natural backgrounds (performances are the ratio of correct responses). The dashed, horizontal line shows the chance level and each curve represents the performance of a model at different levels of variation, specified with different colors in the right inset. The bold black curve illustrates human performance. The color-coded circle points at the top of each plot, inside the rectangular box, exhibits the *p*-values for comparisons between human and each model obtained by Wilcoxon signed-rank test (for example the performance of the HMAX model was compared to the human in each level of variation separately. The result of comparison for each model in each level provides us with a *p*-value. *P*-values are reported with different colors). The color-coded circle points at the right insets, inside the square boxes, show the *p*-values for all possible comparisons for human responses in different levels of variation (with plain and natural background). Here, the *p*-values show whether human categorization performances are significantly different at different levels of variation. For example, we compared the performance of human in Level 0, with Level 1, Level 2, and so on and reported a *p*-value for each comparison. These comparisons resulted in a symmetric *p*-value matrix with the size of 7^*^7 (i.e., 7 levels of variations). **(B)** Performance comparison when objects were presented on plain backgrounds. In both panels **(A,B)**, the results are the average of 15 independent random runs and the error bars show the standard deviation of the mean.

To evaluate the performance of the models, we first randomly selected 300 images from each object category and level (e.g., 300 car images with level one variation). Images were then randomly divided to test and train images. We selected 150 images for the training set and 150 for the test set. All images were converted into grayscale and resized to 200 pixels in height while aspect ratio was preserved. For the case of natural background, we randomly selected equal number of natural images (i.e., 300 images) and superimposed the objects images on these backgrounds. We then fed each model with the images and the performance of each model was obtained for various levels of variation separately. The feature vectors of each model were fed to a linear SVM classifier. The reported results are the average of 15 independent random runs and the error bars are standard deviation of the mean (SD-Figures 3,4,**6**).

Furthermore, the confusion matrices for all models as well as humans were computed in all levels for both plain and natural backgrounds (for multiclass object classification). To obtain a confusion matrix, we first trained a classifier for each category. Then, using these trained classifiers, we computed multiclass performances as well as errors made in classification. To construct a confusion matrix for a given level, we calculated the percentage of classification performance (predicted labels) obtained by each classifier which was trained on a particular category. Confusion matrices can help us to examine which categories are more mistakenly classified. We can also see whether errors increase in high levels of variation.

## Results

### Two-class invariant object categorization

In this experiment, we compared the categorization performance of different models in invariant object recognition tasks with each other and with the categorization performance of human observers. The categorization performance of human observers was measured in psychophysical experiments where subjects were presented with images in different levels of variation. To evaluate the performance of models, we ran similar categorization tasks in which two groups of object categories were selected to perform a two-class object categorization. In the first group, motorcycle and car images were selected, which are both vehicles. For the second group, we selected more dissimilar categories, car and animal images. There were two different types of animal images in this category (i.e., elephant and dinosaur) with variety of examples for each type. We selected 150 images for the training set and 150 for the testing set (see Materials and Methods). The categorization performance of each model was obtained for all levels of variation separately (i.e., seven levels of variation). Figures 3, 4 show the performances of different models as well as human observers in the seven levels of object variation. The results for the deep ConvNet are shown in Figure S3, and are explained in Supplementary Material.

Figure 3 shows the results of animal vs. car classification with natural (Figure 3A) and plain (Figure 3B) backgrounds. In the case of plain background, models performed as accurate as humans in the first two or three levels of variation. Even the Pixel model, which gray values of images were directly fed into the classifier, performed very close to humans in the first two levels of variation. From the level three onward, the performance of the two null models (i.e., V1-like and Pixel) decreased sharply down to 60% in the last level of variation (note that chance level is 50%). Likewise, from the third level up to the sixth level, the performances of other models diminished significantly compared to humans. This shows that the models fail to solve the problem of invariant object recognition when the level of variation grows up. Comparing the performances of the V1-like model and the Pixel model shows that the V1-like model has slightly better invariant responses than the Pixel model. In more complex variations, four other hierarchical models, which implement the hierarchical processing from V1 to V4 and aIT, exhibited higher performances, compared to the null models. Nevertheless, in high levels of variation, even the cortex-like hierarchical models performed significantly lower than human subjects.

Interestingly, when objects are presented on plain backgrounds, the categorization performance of humans in any level of image variation is not significantly different from other levels (see *p*-values in Figure 3 bottom right inset). This means that human observers, as opposed to the models, were able to produce equally well invariant representations in response to objects under different levels of image variation. Indeed, the models are still far below the performance of humans in solving the problem of invariant object recognition (see *p*-values for all comparisons between the models and human observers at the top inset in Figure 3, specified with color-coded circle points inside the rectangular box).

We also compared the performance of the models with humans in a more difficult task, in which objects were presented on randomly selected natural backgrounds instead of plain backgrounds (Figure 3A). A natural background makes the task more difficult for models as well as for humans. In this case, overall, there is a significant difference between the categorization performance of the models and human, even in zero level variation (i.e., no variation, Level 0). In the last three levels of variation (i.e., Levels 4–6), we can see a decrease in human categorization performance (see the *p*-values at the bottom right inset in Figure 3). Although adding natural backgrounds diminished the performance of human in invariant object recognition, the human responses are still robust to different levels of variations and still significantly higher than the models (see *p*-values for all comparisons between the models and human at the top inset in Figure 3).

The lower performances of models in the case of natural backgrounds in comparison to the plain backgrounds show that the feedforward models have difficulties in distinguishing a target object from a natural background. Natural backgrounds impose more complexity to object images and the process of figure-ground segregation becomes more difficult. Studies have suggested that recurrent processing is involved in figure-ground segregation (Roelfsema et al., 2002; Raudies and Neumann, 2010). This may explain why we observe a dramatic decrease in the categorization performance of feedforward models in the natural background condition. They lack a figure-ground segregation step that seems to arise from feedback signals.

Figure 3 shows the categorization performances for car vs. animal images, which are two dissimilar categories, across different levels of variations. To evaluate the performances of human and models in categorizing two similar categories, we used car and motorcycle images, which are both vehicles with similar properties (e.g., wheels). The results are shown in Figure 4A (with natural background) and Figure 4B (with plain background). Overall, the results in both experiments are similar, except that the performances are lower in car vs. motorcycle categorization task.

**Figure 5:**
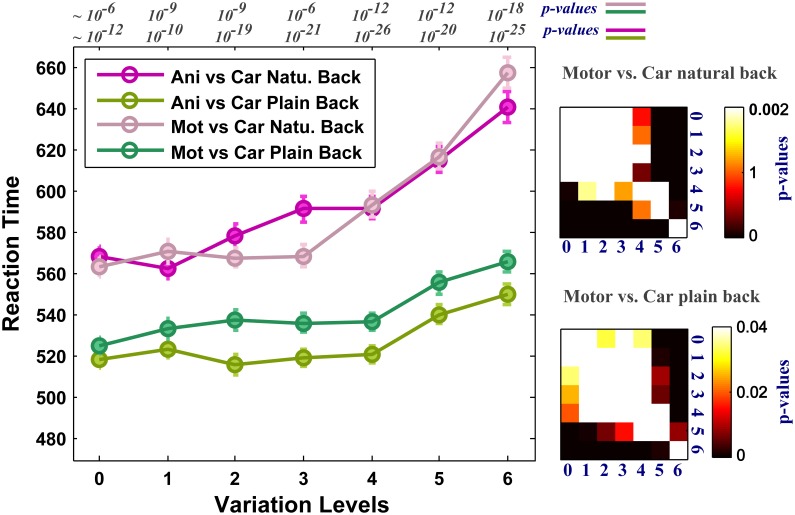
**Human reaction times (RTs) for different levels of variation in two-class invariant object categorization tasks with plain and natural backgrounds**. The RTs were almost equal across all levels of variation when objects were presented on plain backgrounds (except for the higher levels of variation, see *p*-values for all comparisons at the right insets. We made all possible comparisons between RTs across different levels to find out whether the differences between the RTs are statistically significant. Here we only showed matrices for motorcycle vs. car. Animal vs. car gives similar *p*-value matrices). In contrast, when objects were presented on natural backgrounds, the RTs in all levels of variation increased significantly compared to the plain background condition. Error bars are s.e.m. See *p*-values on the top of the figure show comparisons between natural and plain background conditions.

As the level of variation increases the complexity of images grows in both plain and natural backgrounds and the performance decreases. We asked whether the complexity of images affects human RTs in high level of variations. RT is considered as a measure of uncertainty that seems to be associated with the amount of accumulated information required for making a decision about an image in the brain. Figure 5 reports the average RTs across subjects in all seven levels of image variation and the two rapid categorization tasks (animal/car and motorcycle/car) for both plain and natural background conditions. In the case of plain background (green curves), the mean RTs are approximately the same for low and middle levels of variations. On the other hand, when objects are presented with natural backgrounds, human RTs increases more sharply as the complexity of object variations increases. This indicates that the visual system requires more time, in higher levels of variation, to accumulate enough information to reach a reliable decision. This suggests that the brain responds differently to different levels of object variation and the time course of responses depends on the strength of variation. Furthermore, having higher RTs in the natural background condition compared with the plain background condition, suggests that some further processes are going on in the first condition, probably to separate the target object from a distracting natural background.

### Multiclass invariant object categorization

We also compared the models with each other and with human observers in multiclass invariant object categorization tasks (five classes of objects). The confusion matrices for all models as well as humans were computed in all seven levels of object variation in both plain and natural background conditions. Overall, the confusion matrices show that the null models make many more errors while categorizing object classes with intermediate and high level of variations compared to the hierarchical cortex-like models. Moreover, they show that humans accurately categorized object images with only a handful of errors even in higher levels of variation in which the complexity of image variation is higher and it is more likely to perceive two different object images as similar.

**Figure 6:**
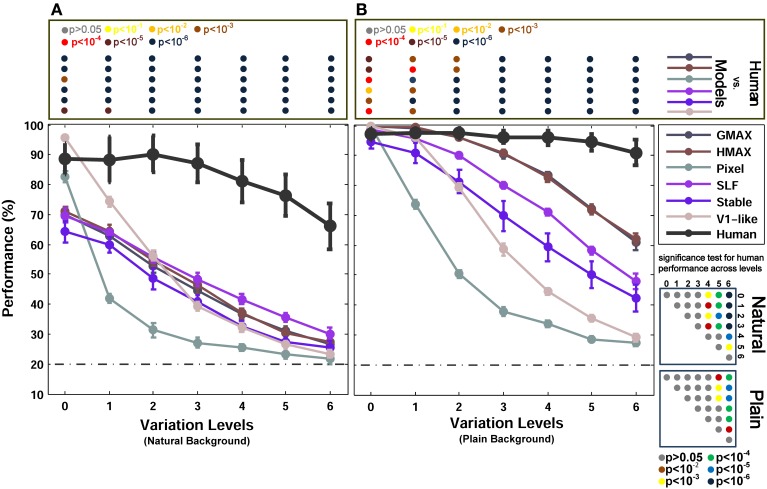
**Performance comparisons between different models and human in multiclass invariant object categorization task. (A)** Performance comparison when objects were presented on natural backgrounds. The dashed, horizontal line shows the chance level (20%) and each curve represents the performance of a model in different levels of variation, specified with different colors in the top right inset. The bold black curve illustrates human performance. The color-coded circle points at the top of each plot, inside the rectangular box, exhibits the *p*-values for comparisons between human and each of the models obtained by Wilcoxon signed-rank test. The color-coded circle points at the right insets, inside the square boxes, show the *p*-values for all possible comparisons for human responses in different levels of variation (with plain and natural background). Here, the *p*-values show whether human categorization performances are significantly different at different levels of variation. For example, we compared the performance of human in Level 0, with Level 1, Level 2, and so on and reported a *p*-value for each comparison. These comparisons resulted in a symmetric *p*-value matrix with the size of 7^*^7 (i.e., 7 levels of variations). **(B)** Performance comparisons when objects were presented on plain backgrounds. In both panels **(A,B)** error bars are STD and the performances are the average of 15 runs.

Figure 6 reports the performances of multiclass object categorization for plain and natural background conditions in all seven levels of object variation. As shown in Figure 6B, when objects were presented on plain backgrounds, all models performed as accurate as humans in zero level variation (no variation-Level 0). In the next level, the performance of the V1-like model was still similar to humans, but it sharply decreased when object images had stronger variations. The performance of the Pixel model dropped dramatically after the zero level variation. This shows that the actual values of pixels do not exhibit an invariant representation. The performances of other models also decreased as the level of image variation increased (from the first level to the last level). In the last level, the performances of the Pixel and V1-like model were very close to the chance level. However, biologically inspired hierarchical models converged on performances higher than chance, although the performances were still much lower than the human performance. Human performances did not significantly differ across different levels of variations, indicating the remarkable ability of human brain in generating invariant representation despite the increasing level of the difficulty in image variations (see *p*-values at the bottom right inset in Figure 6 for all possible comparisons, specified with color-coded circle points inside the square box).

In the case of natural backgrounds (Figure 6A), the performance of the models, even in zero level variation, is significantly lower than the human performance. Interestingly, the V1-like and the Pixel model performed better than other models in zero level variation. This is almost similar to the results reported in Pinto et al. (2008), in which a V1-like model that does not contain any special machinery for tolerating difficult image variations performs better than state-of-the-art models when images have no or very small variations. On the other hand, the representation of these two null models was not informative enough in higher levels of variation and the performance of these models rapidly falls off as the variation gets more difficult (Figure 6A).

**Figure 7:**
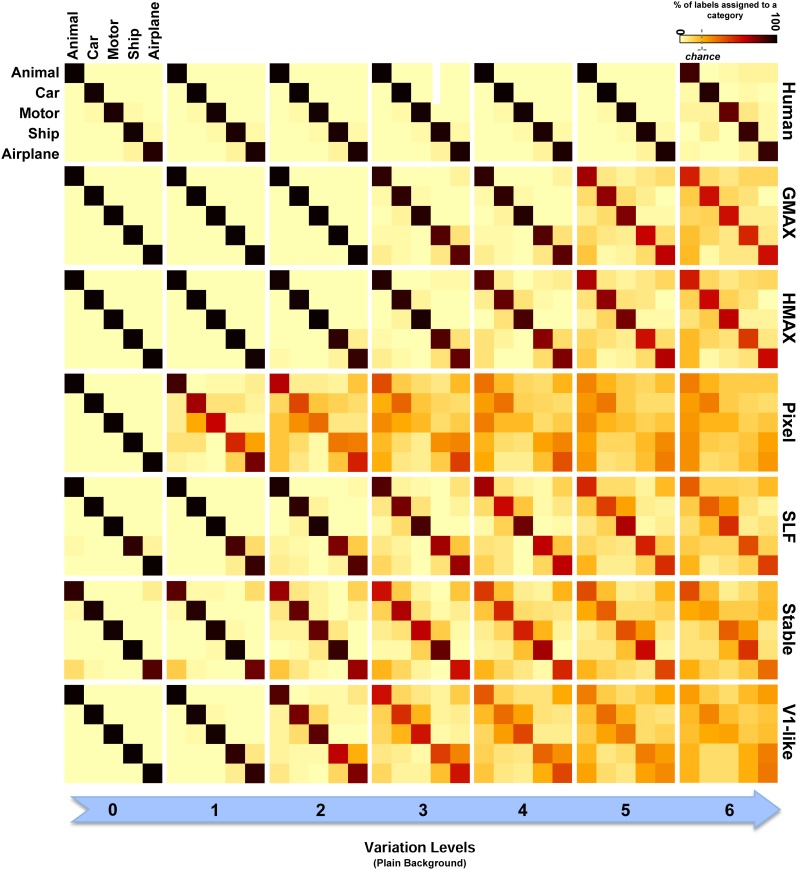
**Confusion matrices for the multiclass invariant object categorization task with plain background**. Each color-coded matrix shows the performance of a model in categorizing different object categories, as specified in the first matrix at the top-left corner. Matrices in each column show confusion matrices for a particular level of variation (from 0 to 6) and each row shows confusion matrices for one model (model name is written at the right end of each row). The first row illustrates the performance of humans in psychophysical experiments. The color bar at the top-right color codes the percentage of the subject responses (labels) assigned to each category. The chance level is specified with a dashed line on the color bar.

**Figure 8:**
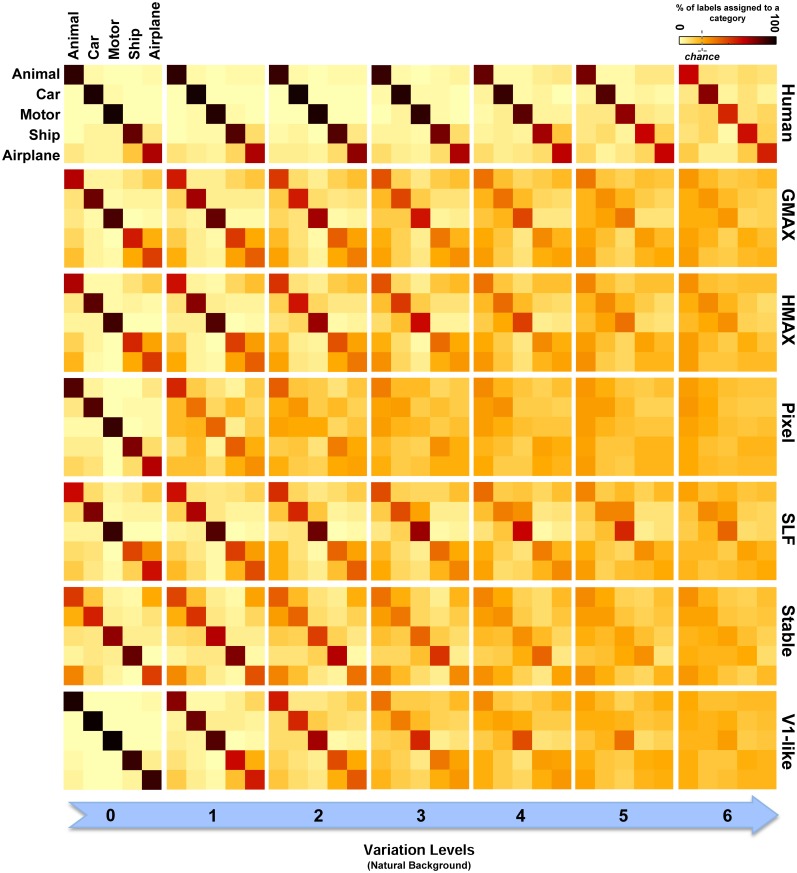
**Confusion matrices for the multiclass invariant object categorization task with natural background**. Each color-coded matrix shows the confusion matrix for a model or for humans in categorizing different object categories presented on natural backgrounds. Matrices in each column show confusion matrices for a particular level of variation (from 0 to 6) and each column shows confusion matrices for one model (model name is written at the right end of each row). The first row illustrates the performance of humans in psychophysical experiments. The color bar at the top-right color codes the percentage of the subject responses (labels) assigned to each category. The chance level is specified with a dashed line on the color bar.

To have a closer look at the performance of humans and models in categorizing each object category and complexity level, we used confusion matrices. Figures 7, 8 show confusion matrices for plain and natural backgrounds, respectively. In the plain background condition, confusion matrices for humans in all levels are completely diagonal that shows the ability of humans in discriminating objects without difficulty, even in higher levels of image variation. The confusion matrices of models are also diagonal in the first two levels of variation. However, models made more errors in higher levels of variation. The Pixel and V1-like models, for example, made many errors in classification of different objects in last levels of variations. This shows that the internal representation of these null models does not tolerate identity-preserving variations beyond a very limited extent. Furthermore, we do not expect responses of V1 neurons to be clustered based on semantic categories (e.g., Kriegeskorte et al., 2008b; Cichy et al., 2014). So a linear readout would not be able to readily decode from V1 responses. This is similar to what we see in the V1 model. Although the representation of V1 neurons are not clustered according to object categories, during recurrent interactions between higher and lower visual areas, early visual areas contribute in categorization and perception happening in higher levels of visual hierarchy (Koivisto et al., 2011). Feedback signals, from higher visual areas toward early visual areas, such as V1, have also been shown to play a role in figure-ground segregation (Heinen et al., 2005; Scholte et al., 2008), which is a useful mechanism in discriminating target objects from cluttered background.

Models made more errors when objects were presented on natural backgrounds (Figure 8). Incorporating object images with randomly selected natural scenes have made the task more difficult for human observers as well. However, the human observers only made a few errors in the last two levels of variation and the confusion matrices for all levels are still close to diagonal. In the models, there are more errors in high and even moderate levels of image variation. As can be seen, the confusion matrices for models are not strongly diagonal in the last two levels of variation. This indicates that models were unable to discriminate objects in higher variations.

**Figure 9:**
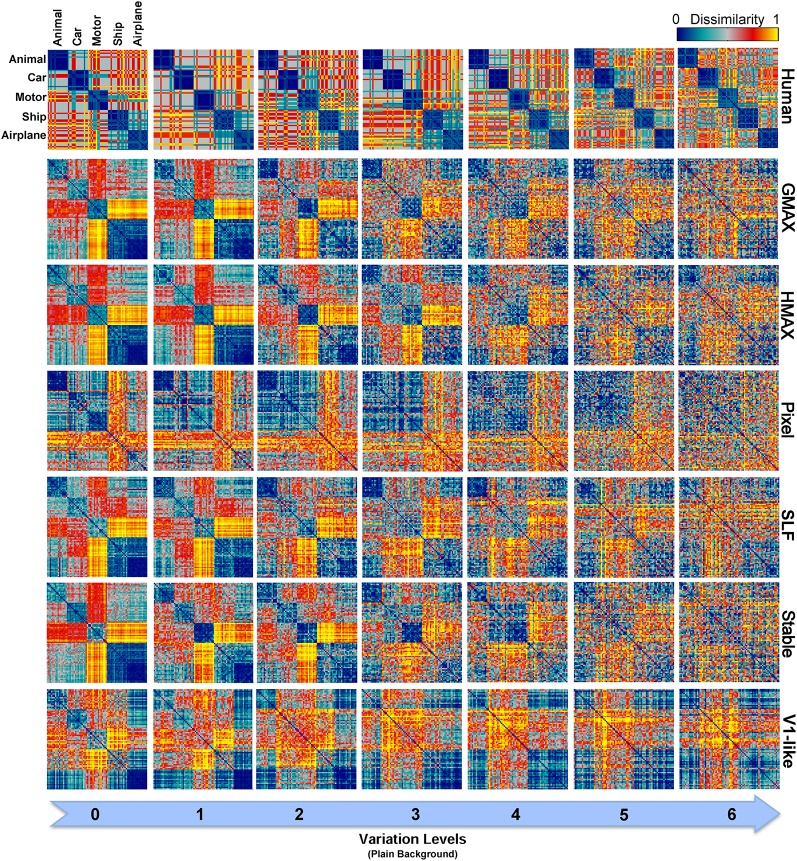
**Representational Dissimilarity Matrices (RDM) for multiclass invariant object categorization with plain background across different levels of variation, calculated based on models' features vector**. Each element in a matrix shows the pairwise dissimilarities between the internal representations of a model for pairs of objects (see Materials and Methods). Each column in the figure shows the RDMs for a particular level of variation (from 0 to 6) and each row shows the RDMs of a model in different levels of variation. The first row illustrates the RDMs for human calculated based on responses in psychophysical experiments. The color bar at the top-right corner shows the degree of dissimilarity (measured as: 1-correlation— Spearman's rank correlation). The size of each matrix is 75^*^75. For visualization, we selected a subset of responses to images in each category (15 images from each category).

In zero level variation, the Pixel and V1-like models achieved performances comparable to human in both cases, plain and natural background (Figures 6A,B). Comparing the internal representation of models gives us further insights about the ability of models in generating identity-preserving invariant representations. To this end, we used RSA (Kriegeskorte et al., 2008a,b) and compared the dissimilarity-patterns of models with each other and with human observers. Figure 9 represents RDMs for different models, calculated directly from feature vectors of each model in seven levels of variation when objects were presented on plain backgrounds. The RDMs for humans are based on the behavioral results, using the labels assigned to each image by human subjects (see Materials and Methods). As can be seen, the dissimilarity representation of models, even in the first levels of variation, does not provide a strong categorical representation for different object classes. However, the RDMs of human show clear clustered representations for different object categories across all levels (first row in Figure 9).

**Figure 10:**
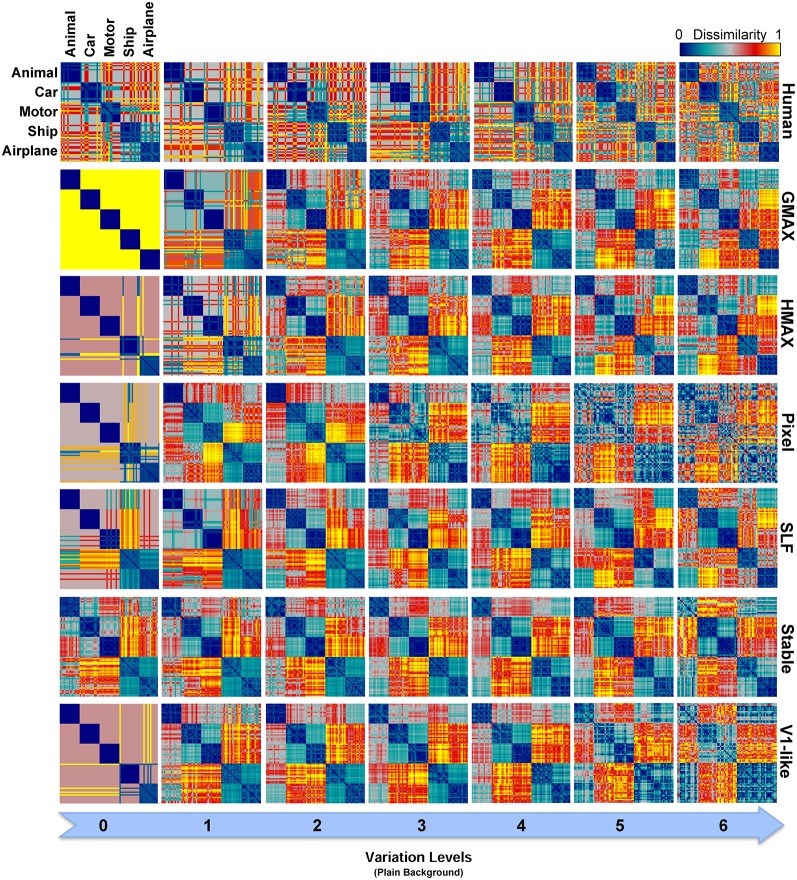
**Representational Dissimilarity Matrices (RDM) for multiclass invariant object recognition with plain background across different levels of variation, obtained based on classifier responses**. Each element in a matrix shows the pairwise dissimilarities between the internal representations of a model for pairs of objects (see Materials and Methods). Each column in the figure shows the RDMs for a particular level of variation (from 0 to 6) and each row shows the RDMs of a model in different levels of variation. The first row illustrates the RDMs for human calculated based on responses in psychophysical experiments. The color bar at the top-right corner shows the degree of dissimilarity (measured as: 1-correlation—Spearman's rank correlation). The size of each matrix is 75^*^75. For visualization, we selected a subset of responses to images in each category (15 images from each category).

As described earlier, the human RDMs were built based on the labels given to the presented images while the RDMs of the models calculated using model features. For further comparisons and to make human RDMs more comparable to models' RDMs, we similarly constructed RDMs for models based on the classifier outputs. Figure 10 illustrates the RDMs of the models based on the SVM responses for the case of objects presented on plain backgrounds. Visual inspection shows that the representations of several models are comparable to humans in different levels of variation. This simply indicates that classifier performs well in categorizing different object categories with high and intermediate levels of variation. However, this similarity structure significantly reduces when models were presented with objects on natural backgrounds (Figure S2 in Supplementary Materials).

As can be seen from RDMs in Figures 9, 10, some object categories (i.e., ship and airplane) have more similar representations in the model space compared to other categories. Interestingly, this can also be seen in the confusion matrices of the models as well as the confusion matrices of human observers (Figures 7, 8). This effect is clearer in Figure 8. These results suggest that the observed similarities are mainly driven by the shape similarly of objects (both ship and airplane share similar shape properties such as body, sail, and wing, etc.). This result was expected for the models since the models were all unsupervised models, and therefore by definition the extracted features were only aware of the shape similarity between the objects and had no additional cue about their category labels. But, human observers similarly made more errors in categorization of these two categories indicating the role of shape similarity in object recognition (Baldassi et al., 2013).

**Figure 11:**
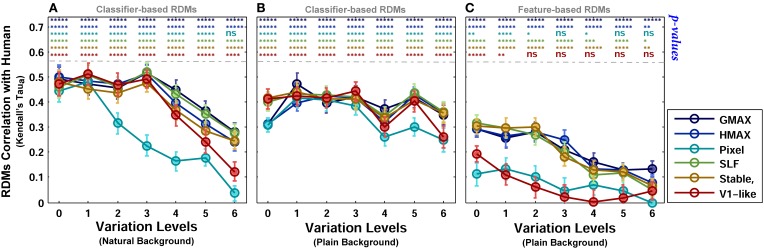
**Correlation between human and model RDMs in different background conditions and complexity levels. (A)** Correlation between human RDMs and model RDMs across different levels of variation, calculated based on classifier responses, when objects were presented on natural backgrounds. **(B)** Correlation between human RDMs and model RDMs across different levels of variation, obtained based on classifier responses, when objects were presented on plain backgrounds. **(C)** Correlation between human RDMs and model RDMs across different levels of variation, obtained based on models' feature vector, when objects were presented on plain backgrounds. The *p*-values for correlation between human and each of the models are shown at the top of each plot, specified with different colors for different models (ns, means not significant; ^*^p < 0.05; ^**^p < 0.005; ^****^p < 10^−4^; and ^*****^p < 10^−6^). Error bars are standard deviations of the mean. Correlation results are the average over 10,000 bootstrap resamples (we used Kendall tau-a rank correlation). The RSA tool box was used for correlation calculation (Nili et al., 2014).

To provide a quantitative measure for better comparisons between human and models, we computed the correlation between each model RDM and human RDM in different levels of variation (Kendall tau-a rank correlation). Figure 11 shows the correlation between the models and human in different complexity levels and conditions (i.e., plain and natural background). The highest correlation among all is close to 0.5. The correlation between the human RDMs and model RDMs, calculated based on model features, is lower compared to RDMs obtained based on the classification responses (Figure 11C). After classification, the responses of several models in different levels are more correlated with human responses, Figures 11A,B.

## Discussion

### Humans perform significantly better than models in discriminating objects with high level of variations

Humans are very fast in categorizing natural images and different object categories (e.g., Potter and Levy, 1969; Thorpe et al., 1996; Vanrullen and Thorpe, 2001; Fabre-Thorpe, 2011). Behavioral studies have demonstrated that humans are able to identify ultra-rapidly presented images from different object categories (Kirchner and Thorpe, 2006; Mack and Palmeri, 2011; Potter et al., 2014). These studies indicate that feedforward visual processing is able to perform a great deal of different visual tasks, although limited to a certain extent (Kreiman et al., 2007; Fabre-Thorpe, 2011). Using psychophysical experiments, we showed that humans are able to remarkably perform invariant object recognition with high performance and minimum time. Although the similarity between two different views of the same object is much lower than the similarity between two different objects (Cox, 2014), human observers could accurately and quickly discriminate different objects categories in different complexity levels (both in two- and multiclass rapid categorization tasks). This task is of immense difficulty for models with many false alarms due to lack of selectivity-invariance trade-off and some other mechanisms, such as figure-ground segregation in cluttered images. Considering the RTs and categorization performances of human observers in the two-class rapid object categorization experiments, we saw that humans were able to respond accurately and swiftly to rapidly presented images with different levels of complexity either when objects were presented on plain backgrounds or on natural backgrounds. This contrasts with the categorization performance of models where they performed weakly in high and intermediate levels of image variation. Further explorations of the errors made in multiclass invariant object recognition, analyzed using confusion matrices, demonstrated that the error rate of the models in categorization was increased in accordance with the complexity of image variations. However, human accuracy remained high even in complex image variations; and humans performed significantly better than the models in categorizing different objects in all seven levels of image variation while objects were only presented for 25 ms.

### Not all image variations yield the same difficulty for the visual system

Brain responds differently to different types of object variations. For example, size invariant representation appears earlier than position (Isik et al., 2013). This invariant representation of objects is evolved across the ventral visual hierarchy (e.g., Isik et al., 2013; Yamins et al., 2014). An important, yet unanswered, question is whether different types of variations need different processing times and which one is more difficult to solve? From a modeling viewpoint, 3D variations (i.e., rotation in-depth and in-plane) are thought to be more difficult than others (Pinto et al., 2011). However, there are very few studies addressing this problem using real-world naturalistic objects with systematically controlled variations (e.g., see Pinto et al., 2008; Yamins et al., 2014). To reach this goal, we need to explore the behavioral and neural responses to different types of variations applied to real-world objects.

Another question is whether the time course of responses depend on the strength of the variations, the lower the variation, the faster the responses? Here we behaviorally showed that as the complexity level of image variation increases, the performance decreases and the RT increases. This suggests that the responses depend on the strength of variations. One potential future research would be measuring the neural responses to the strength of variations using different recording tools (e.g., EEG/MEG, fMRI and electrophysiology—e.g., Yamins et al., 2014) in different species. It would also be interesting to look at the extent to which feedforward pathway can solve invariant object recognition and whether the visual system requires prolonged exposure of object images and a supervised learning to learn invariance.

### Models are missing a figure-ground segregation step

We observed a significant increase in human RTs when objects were presented on natural backgrounds compared to plain backgrounds (Figure 5, pink curves compared to green curves). This suggests that some further ongoing processes occur when objects have cluttered natural backgrounds. To detect a target in a cluttered background, visual system needs to extract the boarder of the target object (object contours). This process is performed by the mechanism of figure-ground segregation in the visual cortex (Lamme, 1995). Grouping a set of collinear contour segments into a spatially extended object requires sufficient time (Roelfsema et al., 1999), even in plain background. This task is more difficult and time consuming when objects are presented in cluttered natural backgrounds. Therefore, the increase in RTs in the case of natural backgrounds could be due to the time needed for figure-ground segregation (Lamme et al., 1999; Lamme and Roelfsema, 2000).

Studies also suggest that recurrent processing is involved in figure-ground segregation (Roelfsema et al., 2002; Raudies and Neumann, 2010). This may explain why we observe a dramatic decrease in the categorization performance of the feedforward models in the natural background condition. The models are missing a figure-ground segregation step that seems to arise from interlayer and between layers feedback signals.

### The role of feedback and future modeling insights

As studies show, if models can represent object categories similar to IT, they can achieve higher performances in object categorization (Khaligh-Razavi and Kriegeskorte, 2013). Moreover, the timing of several studies indicates that feedback projections may strengthen the semantic categorical clustering in IT neural representations–where objects from the same category, regardless of their variations, are clustered together (Kiani et al., 2007; Kriegeskorte et al., 2008b; Carlson et al., 2013). Therefore, considering the role of feedback in models may lead to better categorization performances when image variation is high.

Recurrent processing can play a pivotal role in object recognition and can help the visual system to make responses that are more robust to noise and variations (Lamme and Roelfsema, 2000; Wyatte et al., 2012; O'Reilly et al., 2013). Having said that, the results of our behavioral experiments demonstrated that even with very fast presentation of images with different levels of variations, human observers perform considerably well. One explanation is that the high categorization performances are not simply the results of initial responses in higher visual areas due to the feedforward sweep. Indeed early category-related responses, which emerge at about 150 ms after stimulus onset, may already involve recurrent activity between higher and lower areas (Koivisto et al., 2011). Another explanation could be that the IT representational geometry in this condition is not strongly categorical—this can be tested with fMRI in future studies—and so object categories are not linearly separable, but perhaps in later stages of the hierarchy (i.e., in PFC) the categorical representation gets stronger, which allows subjects to perform well. It would be interesting to investigate whether a linear read-out can decode the presented objects from the IT representation when recurrent processing is disrupted. Understanding the role of feedforward vs. recurrent processing in invariant object recognition opens a new avenue toward solving the computational crux of object recognition.

### Future directions for understanding how/when/where the invariant representation emerges across the hierarchy of human visual system

It is of great importance to investigate not only where the categorical information emerges in the ventral visual pathway (Henriksson et al., 2013), but also when the representations of stimuli in the brain reaches to a level that shows categorical information clearly (Cichy et al., 2014). Having accurate temporal and spatial information of object representation in the brain can help us to know where the invariant representations emerge and how long it takes to have sufficient information about them. This can help us to understand how neural representations evolve over time and different stages in the ventral visual system that finally result in this remarkable performance in invariant object recognition without losing specificity to distinguish between similar exemplars. Moreover, it opens new ways for developing models that have similar representations and performance to the primates' brain (Yamins et al., 2014).

We need to exploit new recording technologies, such as high-resolution fMRI, MEG, and cutting-edge cell recording, to simultaneously record large population of neurons throughout the hierarchy, and advanced computational analyses (Kriegeskorte et al., 2008b; Naselaris et al., 2011; Haxby et al., 2014) in order to understand the mechanisms of invariant object recognition. This would help us to understand when and where invariant responses emerge in response to naturalistic object images with controlled image variations such as our database.

### Conflict of interest statement

The authors declare that the research was conducted in the absence of any commercial or financial relationships that could be construed as a potential conflict of interest.

## Supplementary material

### Deep supervised convolutional neural network vs. humans

In addition to the models we discussed in the paper, we also tested a recent deep supervised convolutional network (Krizhevsky et al., 2012) that has been shown to be successful in different object classification tasks. The model is trained with extensive supervision (over a million labeled training images).

Given that all the feedforward models discussed so far failed to reach human level performance in higher levels of image variation, we were interested to see how a deeper feedforward model that is supervised with more training images will perform in our invariant object recognition task. Similar to other experiments, we compared the model performance against humans in two binary (animal vs. car and motorcycle vs. car) and one multiclass invariant object categorization tasks, both with plain and natural background. The results show that in high image variations humans perform significantly better than the model (Figure S3). Particularly, in all tasks, when the image variation is 4 or higher, humans are always better.

Figure S1 **Sample images in different levels of variation with natural backgrounds**. Object images, rendered from 3D planes, vary in four dimensions: size, position (x, y), rotation in-depth, and rotation in plane, superimposed on randomly selected natural background.

Figure S2 **Representational Dissimilarity Matrices (RDM) for multiclass invariant object categorization task with natural background across different levels of variations, obtained based on classifier responses**. Each element in a matrix shows the pairwise dissimilarities between the internal representations of a model for pairs of objects (see Materials and Methods). Each column in the figure shows the RDMs for a particular level of variation (from 0 to 6) and each row shows the RDMs of a model in different levels of variation. The first row illustrates the RDMs for human calculated based on responses in psychophysical experiments. The color bar at the top-right corner shows the degree of dissimilarity (measured as: 1-correlation- Spearman's rank correlation). The size of each matrix is 75^*^75. For visualization, we selected a subset of responses to images in each category (15 images from each category).

Figure S3 **The performance of the Deep Convolutional Neural Network (DCNN) in invariant object categorization tasks. (A)** Performances in animal vs. car categorization task across different levels of variation. Black curve shows human performance and green curve shows the performance of DCNN. The top plot illustrates the performances when objects were presented on plain backgrounds and the bottom plot shows the performances when objects were presented on natural backgrounds. *P*-values for comparisons between human and the model across different levels of variation are depicted at the top of each plot (Wilcoxon signed-rank test). **(B)** Performances in motorcycle vs. car invariant categorization task across different levels of variation. The top plot illustrates the performances when objects were presented on plain backgrounds and the bottom plot shows the performances when objects were presented on natural backgrounds. *P*-values for comparisons between human and the model across different levels of variation are depicted at the top of each plot (Wilcoxon signed-rank test). The results are the average of 15 independent random runs and the error bars show the standard deviation of the mean. **(C)** Performance comparisons between DCNN and human in a multiclass invariant object recognition task. Left plot shows the performance comparison when objects were presented on plain backgrounds while the right plot shows the performances when objects were presented on natural backgrounds. **(D)** Representational Dissimilarity Matrices (RDM) for DCNN in multiclass invariant object recognition with plain (left column) and natural (right column) background across different levels of variation, calculated based on models' feature vector. Each element in a matrix shows pairwise dissimilarities between the internal representations of the model for pairs of objects. The color bar at the top-right shows the degree of dissimilarity (measured as: 1-correlation- Spearman's rank correlation). For visualization, we selected a subset of responses to images in each category (15 images from each category), meaning that the size of each matrix is 75^*^75. **(E)** Correlation between human and DCNN RDMs (based on DCNN model features from the last layer of the model) in different background conditions and complexity levels. Correlations across all levels are significant (^*****^*p* < 10^−6^). Error bars are standard deviations of the mean. *P*-values are obtained by bootstrap resampling the images. The correlation results are the average over 10,000 bootstrap resamples (we used Kendall tau-a rank correlation).
