# Supplementary figures and images for: Feedforward object-vision models only tolerate small image variations compared to human
Source: Front Comput Neurosci. 2014 Jul 18;8:74. doi: 10.3389/fncom.2014.00074 (PMC4103258; doi:10.3389/fncom.2014.00074)

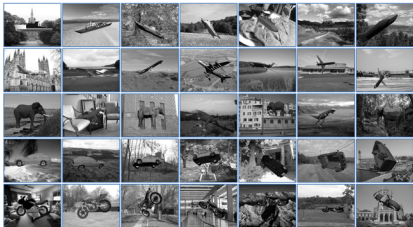

0

1

2

3

4

5

6

Variation Levels

Supplement: Figure S1 — Sample images in different levels of variation with natural backgrounds. Object images, rendered from 3D planes, vary in four dimensions: size, position (x, y), rotation in-depth, and rotation in plane, superimposed on randomly selected natural background. [file Presentation1.PDF]

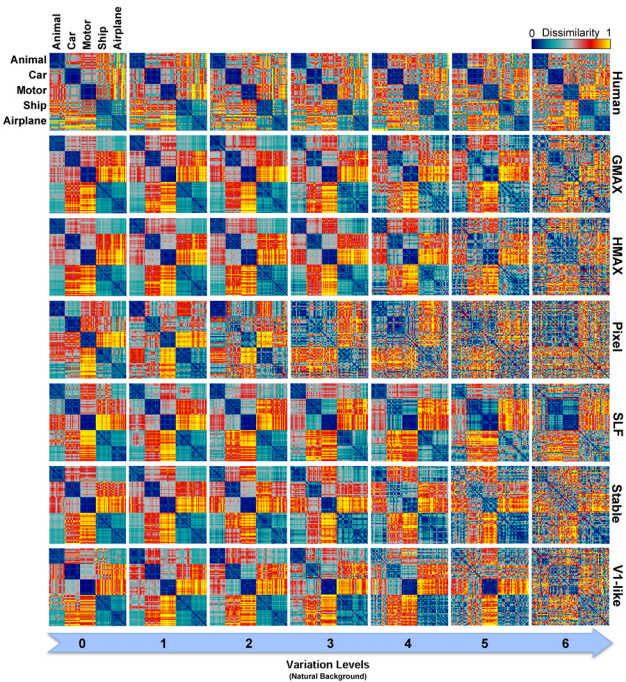

Supplement: Figure S2 — Representational Dissimilarity Matrices (RDM) for multiclass invariant object categorization task with natural background across different levels of variations, obtained based on classifier responses. Each element in a matrix shows the pairwise dissimilarities between the internal representations of a model for pairs of objects (see Materials and Methods). Each column in the figure shows the RDMs for a particular level of variation (from 0 to 6) and each row shows the RDMs of a model in different levels of variation. The first row illustrates the RDMs for human calculated based on responses in psychophysical experiments. The color bar at the top-right corner shows the degree of dissimilarity (measured as: 1-correlation- Spearman's rank correlation). The size of each matrix is 75*75. For visualization, we selected a subset of responses to images in each category (15 images from each category). [file Presentation2.PDF]

**A.**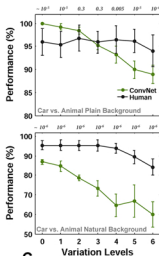**B.**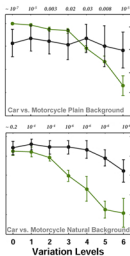**D.**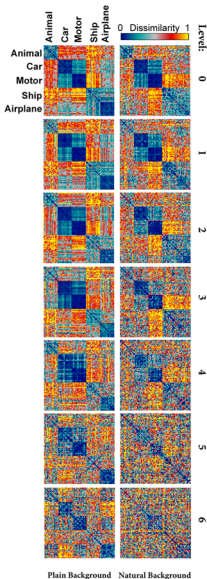**C.**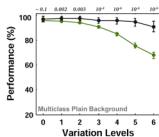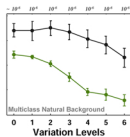**E.**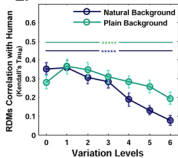

Supplement: Figure S3 — The performance of the Deep Convolutional Neural Network (DCNN) in invariant object categorization tasks. (A) Performances in animal vs. car categorization task across different levels of variation. Black curve shows human performance and green curve shows the performance of DCNN. The top plot illustrates the performances when objects were presented on plain backgrounds and the bottom plot shows the performances when objects were presented on natural backgrounds. P-values for comparisons between human and the model across different levels of variation are depicted at the top of each plot (Wilcoxon signed-rank test). (B) Performances in motorcycle vs. car invariant categorization task across different levels of variation. The top plot illustrates the performances when objects were presented on plain backgrounds and the bottom plot shows the performances when objects were presented on natural backgrounds. P-values for comparisons between human and the model across different levels of variation are depicted at the top of each plot (Wilcoxon signed-rank test). The results are the average of 15 independent random runs and the error bars show the standard deviation of the mean. (C) Performance comparisons between DCNN and human in a multiclass invariant object recognition task. Left plot shows the performance comparison when objects were presented on plain backgrounds while the right plot shows the performances when objects were presented on natural backgrounds. (D) Representational Dissimilarity Matrices (RDM) for DCNN in multiclass invariant object recognition with plain (left column) and natural (right column) background across different levels of variation, calculated based on models' feature vector. Each element in a matrix shows pairwise dissimilarities between the internal representations of the model for pairs of objects. The color bar at the top-right shows the degree of dissimilarity (measured as: 1-correlation- Spearman's rank correlation). For visualization, w [file Presentation3.PDF]
